# SMARCB1/INI1-deficient tumors of adulthood

**DOI:** 10.12688/f1000research.24808.2

**Published:** 2020-12-09

**Authors:** Nathaniel A. Parker, Ammar Al-Obaidi, Jeremy M. Deutsch

**Affiliations:** 1 University of Kansas School of Medicine, 1010 N Kansas St, Wichita, KS, 67214, USA; 2 Cancer Center of Kansas, 818 N. Emporia #403, Wichita, KS, 67214, USA

**Keywords:** SMARCB1, INI1, loss of function mutation, rhabdoid, sarcoma

## Abstract

The *SMARCB1/INI1* gene was first discovered in the mid-1990s, and since then it has been revealed that loss of function mutations in this gene result in aggressive rhabdoid tumors. Recently, the term “rhabdoid tumor” has become synonymous with decreased *SMARCB1/INI1* expression. When genetic aberrations in the *SMARCB1/INI1* gene occur, the result can cause complete loss of expression, decreased expression, and mosaic expression. Although SMARCB1/INI1-deficient tumors are predominantly sarcomas, this is a diverse group of tumors with mixed phenotypes, which can often make the diagnosis challenging. Prognosis for these aggressive tumors is often poor. Moreover, refractory and relapsing progressive disease is common. As a result, accurate and timely diagnosis is imperative. Despite the *SMARCB1/INI1* gene itself and its implications in tumorigenesis being discovered over two decades ago, there is a paucity of rhabdoid tumor cases reported in the literature that detail *SMARCB1/INI1* expression. Much work remains if we hope to provide additional therapeutic strategies for patients with aggressive SMARCB1/INI1-deficient tumors.

## History of the *SMARCB1/INI1* Gene

SWI/SNF-related matrix-associated actin-dependent regulator of chromatin subfamily B member 1 (SMARCB1), also known as integrase interactor 1 (INI1), is a crucial component of a chromatin-remodeling protein complex. *SMARCB1/INI1* was first identified in yeast in the late 1980s ^1^. By 1994, its human homologue was isolated in fibroblast cells ^2, 3^. Subsequent molecular investigations showed this nuclear protein complex enhances DNA transcription by interactions with HIV-1 integrase ^2^. Nuclear *SMARCB1/INI1* exists ubiquitously in all normal cells, and acts as a tumor suppressor gene ^4^. It was revealed in the early 2000s by studies in mice that biallelic knockout of the *SMARCB1/INI1* gene resulted in early lethality ^5^. Mice with heterozygous loss before birth, or who had later conditional single-allele knockout after birth, of *SMARCB1/INI1* developed aggressive rhabdoid tumors ^6– 8^. Since its discovery, much work has revealed this chromatin-remodeling protein has crucial roles in multiple signaling pathways that function to suppress tumorigenesis and tumor growth ^9^. Although these pathways are highly complex, the development and use of targeted anti-cancer therapies has practically become ubiquitous for nearly all solid tumors. Thus, continued investigations are needed if we hope to provide additional therapeutic strategies for patients with aggressive SMARCB1/INI1-deficient tumors ^9^. 

Interestingly, the genetic signatures of SMARCB1/INI1-deficient tumors are far from monotonous. Three distinct patterns of abnormal *SMARCB1/INI1* gene expression have been identified – reduced, complete loss, and mosaic ^9^.

## Epidemiology, clinical, prognosis

Complete loss of *SMARCB1/INI1* expression has been linked to a number of pediatric and adult sarcomas ( Table 1). Malignant rhabdoid tumor (MRT) and epithelioid sarcoma (ES) both result from biallelic deletions or mutations causing a complete loss of *SMARCB1/INI1* expression ^41^. Commonly arising before the age of three years old, MRTs are considered one of the most aggressive childhood neoplasms associated with high mortality ^41^. MRTs have been reported in adults ^42– 49^. Based on MRT of adulthood being primarily reported anecdotally, estimated rates of incidence remain unclear. Data concerning the 5-year survival rate for MRT in adults is difficult to determine as well, as various percentages have been reported in literature ^13, 14^. However, estimated average survival following MRT diagnosis has been reported to be six months ^10^.

**Table 1.** Epidemiologic, selected clinical, and prognostic data for SMARCB1/INI1-deficient tumors. STS, soft tissue sarcomas; MRT, malignant rhabdoid tumor; CNS, central nervous system; MPNST, malignant peripheral nerve sheath tumor; NF-1, neurofibromatosis type 1; NF-2; neurofibromatosis type 2, GI, gastrointestinal; NA, data not available.

		Epidemiology, Clinical	Survival
Reduced expression	Synovial sarcoma	5 – 10% of all STS; median age of 40 years; males ^10, 11^	5-year, 36–76% ^11^
Complete loss	Malignant rhabdoid tumor	Typically < 3 years of age ^11^; typically presents intraabdominally in adult males ^10, 12^	MRT: 5-year, 15 – 20% ^13^; extrarenal rhabdoid tumor: 5-year, 35% ^14^
	Atypical teratoid/rhabdoid tumor	Typically < 3 years of age; 10% of CNS tumors in infants ^12^	20 months ^15, 16^
	Epithelioid sarcoma	< 1% of all STS; median age of 27 years, males ^17^	5-year, 68% (all ages) ^18^
	Renal medullary carcinoma	Third most common kidney cancer among children and young adults; median age of 28 years; males ^19, 20^	Overall survival less than 12 months ^19^
	Epithelioid malignant peripheral nerve sheath tumor	< 1% of all STS; < 5% of all MPNSTs; aggressive MPNST variant; unlike MPNST uncommonly associated with NF-1; median age > 40 years ^21, 22^	5-year, 34 – 43% ^22^
	Myoepithelial carcinoma	About 70% occur in parotid gland; median age of 55 years ^23^	5-year, 71% ^24^
	Extraskeletal myxoid chondrosarcoma	< 3% of all STS; median age of 50 years; males ^25^	5-year, 80 – 90% ^25^
	Chordoma	Median age of 50 – 60 years in adults, males; median age of 10 – 12 years in children, females ^26, 27^	5-year, 70% ^28^
	Pancreas undifferentiated rhabdoid carcinoma	Heterogeneous group of neoplasms; poorly characterized ^29^	NA
	Sinonasal basaloid carcinoma	< 5% of all head/neck cancers; 0.5 cases per 100,000 population per year; males ^30^	Median overall survival 17 months ^31^
	Rhabdoid carcinoma of the gastrointestinal tract	About 0.1% of all gastric cancers; < 50 cases reported in the upper and lower GI tract ^32, 33^	Overall survival six months ^33^
Mosaic expression	Schwannomatosis	Third major form of neurofibromatosis; distinct from NF-1 and NF-2; median age of 40 years; 20% familial ^34^	NA
	Gastrointestinal stromal tumor	5% of all STS, 80% of all mesenchymal GI tract tumors; median age of 60 years ^35– 37^	5-year, 83% ^38^
	Ossifying fibromyxoid tumor	Only 300 cases reported worldwide; median age of 50 years; males ^39, 40^	NA

ES is now categorized into two subgroups: distal and proximal. Conventional or distal-type ES tends to be histologically similar squamous cells. Also, distal-type ES immunohistochemical (IHC) profiles can be diverse. Proximal-type ES is thought to be the more aggressive variant, and has an affinity for the proximal limbs of young adults. Microscopically, sheets of large rhabdoid tumor cells are predominantly observed ^50^. Based on more recent clinicopathologic and IHC data, many tumors that were previously diagnosed as a MRT are now classified as proximal ES ^51^.

In addition to ES, atypical teratoid/rhabdoid tumor, renal medullary carcinoma, and pediatric chordoma are rare sarcomas that result from the complete loss of *SMARCB1/INI1* expression ( Table 1). They predominantly occur in pediatric or young adult patients. Collectively, these neoplasms typically develop in the head/neck, CNS, thorax, kidneys, other visceral organs, retroperitoneum, trunk, and extremities ^12, 17, 19, 26, 52^. Exceedingly rare SMARCB1/INI1-deficient tumors that occur more commonly in adults include synovial sarcomas, epithelioid malignant peripheral nerve sheath tumor, myoepithelial carcinoma, extraskeletal myxoid chondrosarcoma, chordoma, schwannomatosis, gastrointestinal stromal tumors (GIST), and ossifying fibromyxoid tumor ( Table 1). On light microscopy, these sarcomatous neoplasms exist on a morphological spectrum. Tissue specimens are often composed of epithelioid or rhabdoid cells ^53^. However, other morphologic patterns have been described ^50^. Thus, the diagnosis of SMARCB1/INI1-deficient tumors can be difficult based on their polyphenotypic variation ^4^. SMARCB1/INI1 immunostaining can be used to confirm the diagnosis of an epithelioid or rhabdoid sarcoma because loss of *SMARCB1/INI1* expression is rarely observed in other tumor types ^54, 55^. Thus, in the absence of this genetic alteration, other malignant soft tissue tumors with epithelioid-like morphologies can be more confidently ruled out, such as melanoma, rhabdomyosarcoma, and undifferentiated carcinoma.

Aside from SMARCB1/INI1-deficient tumors sharing an aberration in the same gene, the relationship between these malignancies remains unclear. Following diagnosis in any age or organ, nearly all SMARCB1/INI1-deficient malignancies characteristically follow an aggressive clinical pattern and prognosis is often poor ( Table 1). Survival rates are often reportedly low, but they may not be accurate given low rates of incidence, and considerations for newer treatments. Also, survival can be highly dependent on surgical intervention and completeness of tumor resection, especially for chordomas. GIST are the most common sarcomas of the gastrointestinal (GI) tract. They commonly develop in the sixth decade of life and have no gender predominance ^35^. Following the diagnosis of a GIST, survival rates are highly variable and depend on specific biologic characteristics of the tumor, the type of treatment, and the risk of post-treatment recurrence ^36^. 

## Challenges in retrospective data collection for adult cases of SMARCB1/INI-deficient tumors

Recently, the term “rhabdoid tumor” has become synonymous with tumors that harbor loss of function mutations in the *SMARCB1/INI1* gene ^56^. We reviewed the literature and found a paucity of cases reporting *SMARCB1/INI1* genetic aberrations in adult patients with sarcomas. A total of 450 cases of rare sarcomas were found to be described in single case reports, case series, or systematic reviews published between the years 2000 – 2020 ( Table 2) ^57– 92^. This number is likely far lower than the actual accounts of reported sarcoma cases in the literature. However, reports were excluded if it was apparent the case did not meet our inclusion criteria based on the publicly-available title or abstract information. Despite the *SMARCB1/INI1* gene being discovered in the mid-1990s, the majority of previous reports were excluded for not mentioning the tumor’s SMARCB1/INI1-deficiency status. Also, tumor occurrence in the pediatric patient population accounted for multiple exclusions.

**Table 2.** Excluded rare sarcomas in adults reported in single case reports, case series, or systematic reviews, 2000–2020. Exclusion criteria were as follows: 1.) individual patient age could not be confirmed; 2.) pediatric study population (less than 18 years of age); 3.) absence of documentation noting the loss of *SMARCB1/INI1* expression by immunohistochemistry or genetic studies; 4.) intact *SMARCB1/INI1* expression by immunochemistry or genetic studies; and 5.) non-sarcomatous histologic tumor type. PMID, PubMed Central © unique article identifier; GU, genitourinary; PNS, peripheral nervous system; GI, gastrointestinal.

Article	PMID	Cases, no.	Tumor site	Exclusion reason	Article	PMID	Cases, no.	Tumor site	Exclusion reason
Zhang *et al.*, 2019 ^57^	31933781	1	scalp	3	Weisskopf *et al.*, 2006 ^75^	16474944	1	spine	2,3
Kubota *et al.*, 2019 ^58^	31034722	1	GU	2	Onol *et al.*, 2006 ^76^	16343734	1	GU	3
Kolin *et al.*, 2018 ^59^	29700418	5	GU	4	Zevallos-G. *et al.*, 2005 ^77^	16082246	2	perineum	3
Kim *et al.*, 2018 ^60^	30235775	1	brain	3	Masunaga *et al.*, 2004 ^78^	15260853	1	lung	3
Strehl *et al.*, 2015 ^61^	25920939	25	GU	3	Chang *et al.*, 2004 ^79^	14713833	1	GI	2
Santos *et al.*, 2013 ^62^	23793215	1	pelvis	3	Altundag *et al.*, 2004 ^80^	15579921	1	GU	3
Patrizi *et al.*, 2013 ^63^	23886403	1	GU	3	Lee *et al.*, 2004 ^81^	14675288	1	pelvis	3
Zhao *et al.*, 2013 ^49^	23761028	1	renal	3	Peng *et al.*, 2003 ^44^	12946214	1	renal	3
Tocco *et al.*, 2012 ^64^	23359842	1	scalp	3	Hanna *et al.*, 2002 ^82^	12107573	8	multiple	3
Rizzo *et al.*, 2012 ^65^	22614000	12	PNS	2	Etienne-M. *et al.*, 2002 ^83^	12445750	12	multiple	3,5
Kuge *et al.*, 2012 ^66^	22218708	1	brain	2	Moore *et al.*, 2002 ^84^	11925150	1	GU	3
Hagström *et al.*, 2011 ^67^	21420628	1	oral	3	Haidopoulos *et al.*, 2002 ^[Bibr ref-85]^	12440823	1	GU	3
Narendra *et al.*, 2010 ^68^	20479553	1	GU	3	Tzilinis *et al.*, 2002 ^86^	16093195	1	GU	3
Tholpady *et al.*, 2010 ^69^	20881848	1	GU	2	Amrikachi *et al.*, 2002 ^87^	12478486	4	GI	3
Chbani *et al.*, 2009 ^55^	19141382	106	multiple	1	Hasegawa *et al.*, 2001 ^88^	11454997	20	multiple	3
Hornick *et al.*, 2009 ^70^	19033866	127	multiple	1	Kasamatsu *et al.*, 2001 ^89^	11520372	1	GU	3
Kim *et al.*, 2008 ^71^	19471567	1	GU	3	Knapik *et al.*, 2001 ^90^	11521235	1	GU	4
Rekhi *et al.*, 2008 ^72^	18607629	40	multiple	3	Biegal *et al.*, 2000 ^91^	10738300	1	brain	2
Argenta *et al.*, 2007 ^73^	17692365	1	GU	3	Spillane *et al.*, 2000 ^92^	10791853	37	multiple	3
Bourdeaut *et al.*, 2007 ^74^	17152049	26	multiple	2					

We located 25 cases of adult SMARCB1/INI1-deficient sarcomas that were described in 18 reports ( Table 3) ^42, 50, 93– 108^. Median age at the time of diagnosis was 36 years old. A male predominance was mildly observed (14 cases, 56%), which is consistent with other larger reviews. Presentation in the head and neck (e.g. brain, eye, nose, and scalp) occurred more frequently (6 cases, 24%). No descriptive data analysis was performed to determine if our observations were significant. The majority of reports were originally described as proximal epithelioid sarcoma, but overall these remained a morphologically diverse group of cases that also included rhabdoid and mixed phenotypes.

**Table 3.** Included rare sarcomas reported in single case reports, case series, or systematic reviews, 2000–2020. Inclusion criteria were as follows: ability to confirm an individual case patient was greater than 18 years of age; documentation of a loss of *SMARCB1/INI1* expression by immunohistochemistry or genetic studies; and confirmed sarcomatous histologic tumor type. “ - “ denotes complete, reduced, or mosaic loss of *SMARCB1/INI1* expression (exp.). M, male; F, female.

Article	PMID	Cases, no.	Age, Sex	Tumor site	SMARCB1/ INI1 exp.	Sarcoma morphology
Parker *et al.*, 2020 ^42^	32467817	1	56 M	inguinal	-	epithelioid, rhabdoid
Ahmad *et al.*, 2019 ^93^	31737506	1	27 M	pleura	-	epithelioid
Bodi *et al.*, 2018 ^94^	29541486	1	22 F	brain	-	epithelioid, spindle-shaped
Gurwale *et al.*, 2017 ^95^	-	1	18 F	scalp	-	epithelioid
Saha D *et al.*, 2016 ^96^	27045049	1	41 M	lung	-	epithelioid
Rego *et al.*, 2015 ^97^	25737787	1	34 F	vulva	-	epithelioid, spindle-shaped
Wetzel *et al.*, 2014 ^98^	24997629	1	51 F	oral	-	rhabdoid
Agaimy *et al.*, 2014 ^99^	24503755	1	66 M	stomach	-	rhabdoid
Madsen *et al.*, 2013 ^100^	24457248	1	45 M	pleura	-	epithelioid
Frank *et al.*, 2013 ^101^	24308011	1	43 M	eye	-	epithelioid, spindle-shaped
		2	71 F	nasal	-	epithelioid
Kim *et al.* 2012 ^102^	21724432	1	41 F	vulva	-	epithelioid
Mannan *et al.*, 2010 ^103^	19757197	1	47 M	inguinal	-	epithelioid
Takei *et al.*, 2010 ^104^	19911885	1	33 F	brain	-	rhabdoid
		2	79 M	cecum	-	rhabdoid
Raoux *et al.*, 2009 ^105^	19342946	1	31 F	bone	-	epithelioid, spindle-shaped
Robbens *et al.*, 2006 ^106^	16602014	1	19 M	vertebra	-	epithelioid
Sigauke *et al.*, 2006 ^107^	16528370	1	26 M	wrist	-	epithelioid
		2	26 M	lymph	-	epithelioid
Perry *et al.*, 2005 ^108^	15761491	1	29 M	soft tissue	-	spindle-shaped
Modena *et al.*, 2005 ^50^	15899790	1	31 F	thigh	-	epithelioid
		2	47 F	perineum	-	rhabdoid
		3	30 M	spine	-	epithelioid
		4	36 M	spine	-	epithelioid, spindle-shaped
		5	66 F	inguinal	-	epithelioid, rhabdoid

## Treatment

Prior to, and still after, the discovery that SMARCB1/INI1-deficient tumors contribute to the large majority of soft tissue sarcomas, systemic cytotoxic agents have been used to treat this diverse group of neoplasms. Doxorubicin and ifosfamide have remained the mainstay of first-line treatment for advanced disease for the last few decades. Currently, the most widely used regimen for soft tissue sarcomas is termed AIM, which includes Adriamycin (doxorubicin) plus ifosfamide and mesna ^109– 111^. Therapies such as these, and other cytotoxic agents, exhibit intermediate to improved anti-cancer activity, and prolong survival in metastatic soft tissue sarcoma ( Table 4). However, refractory or progressive disease can occur. With the hopes of improving outcomes in patients who develop aggressive sarcomas, multiple new therapies are being introduced. Olaratumab, a monoclonal antibody that targets platelet-derived growth factor alpha and beta (PDGFRA/B), has been approved for first-line therapy in combination with doxorubicin due to improved progression and overall survival in sarcoma patients ^112^. The use of tyrosine kinase-inhibitors (TKIs) has transformed the treatment of advanced GIST. Imatinib, a TKI, as monotherapy is now approved for upfront treatment of metastatic GIST due to improved side effect profiles and outcomes in these patients ^113– 115^. Given its mechanism of action, imatinib is also approved for first-line treatment of the fibrosarcomatous variant of dermatofibrosarcoma protuberans ^116, 117^.

**Table 4.** Approved first-line treatments for sarcomas. ORR, overall response rate; PFS, progression free survival; OS, overall survival; STS, soft tissue sarcoma; GIST, gastrointestinal stromal tumor; D, doxorubicine; I, ifosfamide; P, palifosfamide; E, Evofosfamide; T, trabectedin; O, Olaratumab; G, gemcitabine; Doc, docetaxel; NA, data not available.

Tumor	Drugs	Schedules	ORR (%)	PFS (months)	OS (months)	Reference
STS	Doxorubicin Ifosfamide Evofosfamide Trabectedin Olaratumab	D + I	26	7.4	14.3	128
		D + P	28.3	6	15.9	129
		D + E	28.4	6.3	18.4	130
		D + T	17	5.7	13.3	131
		D + O	18.2	6.6	26.5	112
	Trabectedin	monotherapy	14.8	2.8	NA	132
	Aldoxorubicin	monotherapy	25	5.6	15.8	133
	Amrubicin	monotherapy	13	5.8	26	134
	Gemcitabine Docetaxel	G + Doc	58.6	5.6	14.7	135
	Brostacillin	monotherapy	3.9	1.6	NA	136
GIST	Imatinib	monotherapy	68.1	18	55	113– 115
Angiosarcoma	Paclitaxel	monotherapy	NA	4	8	137

Additional TKIs have recently been introduced, with clinical trial data showing promise for their use in sarcomas. Sunitinib and regorafenib significantly improve overall survival in imatinib-resistant GIST patients ^118^. Pazopanib, a TKI that targets angiogenesis by inhibiting vascular endothelial growth factor receptor, PDGFRA/B, and KIT proto-oncogene, has been shown to improve progression free survival in certain histologic types of sarcoma. This led to its approval for advanced, refractory non-lipomatous sarcoma ^119, 120^. Alveolar sarcomas appear to respond well to anti-angiogenetic sorafenib and cediranib ^121, 122^. In phase II studies tivozanib, which mechanism of action mimics pazopanib, exhibits promising anti-cancer activity in metastatic or nonresectable soft tissue sarcomas ^123^. 

Recently, much work studying the complex mechanisms involved in sarcoma tumorigenesis has revealed the potential for numerous new drug targets. Targeting the mammalian target of rapamycin (mTOR) signaling pathway by serine/threonine kinase inhibition has been widely studied. However, thus far either only equivocal or minor benefits have been shown with the administration of these agents ^124^. In contrast, phase II trial data is reassuring for the future use of palbociclib, a cyclin-dependent kinase 4 and 6 inhibitor approved in breast cancer, for liposarcoma ^125, 126^.

Preliminary data from pre-clinical and phase I/II trials is encouraging for small molecule inhibitors, such as with Murine double minute 2 (MDM2)–antagonists, histone deacetylase inhibitors, and histone methylation inhibitors ^124^. A possible breakthrough in small molecular inhibition is represented by the recent discovery of a specific methyltransferase termed Enhancer of zeste homolog 2 (EZH2) is upregulated in SMARCB1/INI1-deficient tumors ^127^. Given the defining characteristic of SMARCB1/INI1 deficiency in the nearly all soft tissue sarcomas, tazemetostat has emerged as an intriguing compound for its direct inhibition of histone-lysine N-methyltransferase EZH2 ^127, 138^. Another new agent that hopes to improve outcomes for patients with these rare and aggressive SMARCB1/INI1-deficient rhabdoid sarcomas comes from the proteasome inhibitor drug class. Ixazomib selectively targets proteasomes involved in protein anabolism and cellular apoptosis, whose activity is directly enhanced by the transcription factor MYC in *SMARCB1/INI1*-deficient states. Currently, ixazomib plus gemcitabine and doxorubicin is being studied in the phase II trial setting for renal medullary carcinoma ^139, 140^.

## Data availability

### Underlying data

No data are associated with this article.
