# Supplementary material for: SMARCB1/INI1-deficient tumors of adulthood
Source: F1000Res. 2020 Dec 9;9:662. Originally published 2020 Jun 30. [Version 2] doi: 10.12688/f1000research.24808.2 (PMC7968524; doi:10.12688/f1000research.24808.2)
Supplement: Peer review history

# Peer review: Reviewer response for version 2

**DOI:** 10.5256/f1000research.30897.r80638

**Published:** 2021-03-15

**Authors:** Abdulqadir Nashwan

**Affiliations:** 1 Research Scientist, Hamad Medical Corporation (HMC), Doha, Qatar

This is a review article highlighting the recently published case reports on the role of SMARCB1/INI1-deficient tumors of adulthood and available treatment strategies.

 This review summarized important findings and is theoretically based on the current literature.

 The subject is very important to basic oncology practice.

 The paper is well-written and provides useful information for the readers.

Is the review written in accessible language?

Yes

Are all factual statements correct and adequately supported by citations?

Yes

Are the conclusions drawn appropriate in the context of the current research literature?

Yes

Is the topic of the review discussed comprehensively in the context of the current literature?

Yes

Reviewer Expertise:

Translational Oncology, Immunology

I confirm that I have read this submission and believe that I have an appropriate level of expertise to confirm that it is of an acceptable scientific standard.

# Peer review: Reviewer response for version 1

**DOI:** 10.5256/f1000research.27370.r74306

**Published:** 2020-11-05

**Authors:** Conor Patrick Malone

**Affiliations:** 1 Health Service Executive (Ireland), Dublin, Ireland

This article clearly and concisely reviews the role of *SMARCB1/INI1* in rhabdoid tumours, as well as summarising the literature and discussing management options. The structure is good, the language is accessible, and the references are appropriate and comprehensive.

 I would suggest a short conclusion to recap the main points and to ensure that there are clear learning outcomes for readers of varying experience levels.

 Below are minor grammar/punctuation corrections and suggestions:

 "the result can cause reduced, complete loss, and mosaic expression." - this is not clear - I would suggest changing to "the result can cause reduced expression, complete loss of expression, and mosaic expression" ?

 There should be no apostrophe in 1980’s or 2000's, i.e. 1980s and 2000s are correct.

 In Table 1 "typically presents **in** intraabdominally in adult males" the first " **in**" is an error.

 "CNS" is used without expansion/explanation of the acronym.

 "prognosis if often poor" - should read " **is** often poor".

 In Tables 2 and 3 "criteria was as follows" should be " **were** as follows".

 "A male predominance was mildly observed (14 cases, 56%), which is consistent with other larger reviews." - this wording is unclear - suggest "Consistent with other larger reviews, there was a slight male predominance (14 cases, 56%)."

 "is termed AIM, which includes doxorubicin plus ifosfamide and mesna" - explain this more clearly so that the initialism (AIM) makes sense.

 "KIT" is not an acronym/initialism but suggest "KIT proto-oncogene" so that it is clear what it is.

 Explain what "mTOR" and "MDM2" and "EZH2" stand for.

 "as a highly intriguing" - remove the word "highly".

Is the review written in accessible language?

Yes

Are all factual statements correct and adequately supported by citations?

Yes

Are the conclusions drawn appropriate in the context of the current research literature?

Yes

Is the topic of the review discussed comprehensively in the context of the current literature?

Yes

Reviewer Expertise:

Orbital rhabdomyosarcoma.

I confirm that I have read this submission and believe that I have an appropriate level of expertise to confirm that it is of an acceptable scientific standard.
